# A possible neurophysiological correlate of audiovisual binding and unbinding in speech perception

**DOI:** 10.3389/fpsyg.2014.01340

**Published:** 2014-11-26

**Authors:** Attigodu C. Ganesh, Frédéric Berthommier, Coriandre Vilain, Marc Sato, Jean-Luc Schwartz

**Affiliations:** ^1^CNRS, Grenoble Images Parole Signal Automatique-Lab, Speech and Cognition Department, UMR 5216, Grenoble University, GrenobleFrance; ^2^CNRS, Laboratoire Parole et Langage, Brain and Language Research Institute, UMR 7309, Aix-Marseille University, Aix-en-ProvenceFrance

**Keywords:** audiovisual binding, speech perception, multisensory interactions, EEG

## Abstract

Audiovisual (AV) speech integration of auditory and visual streams generally ends up in a fusion into a single percept. One classical example is the McGurk effect in which incongruent auditory and visual speech signals may lead to a fused percept different from either visual or auditory inputs. In a previous set of experiments, we showed that if a McGurk stimulus is preceded by an incongruent AV context (composed of incongruent auditory and visual speech materials) the amount of McGurk fusion is largely decreased. We interpreted this result in the framework of a two-stage “binding and fusion” model of AV speech perception, with an early AV binding stage controlling the fusion/decision process and likely to produce “unbinding” with less fusion if the context is incoherent. In order to provide further electrophysiological evidence for this binding/unbinding stage, early auditory evoked N1/P2 responses were here compared during auditory, congruent and incongruent AV speech perception, according to either prior coherent or incoherent AV contexts. Following the coherent context, in line with previous electroencephalographic/magnetoencephalographic studies, visual information in the congruent AV condition was found to modify auditory evoked potentials, with a latency decrease of P2 responses compared to the auditory condition. Importantly, both P2 amplitude and latency in the congruent AV condition increased from the coherent to the incoherent context. Although potential contamination by visual responses from the visual cortex cannot be discarded, our results might provide a possible neurophysiological correlate of early binding/unbinding process applied on AV interactions.

## INTRODUCTION

Speech perception requires adequate hearing and listening skills, but it is well known that visual information from the face and particularly from lip movements may intervene in the speech decoding process. The first classical evidence for audiovisual (AV) integration in speech perception in normal-hearing subjects concerns the role of lip reading during speech comprehension, with a gain in the AV modality in respect to the audio-only modality particularly in adverse listening conditions (e.g., Sumby and Pollack, 1954; Erber, 1971; Benoît et al., 1994; Grant and Seitz, 2000; Bernstein et al., 2004b). Another classical behavioral example for AV integration is provided by the McGurk effect (McGurk and MacDonald, 1976), in which a conflicting visual input modifies the perception of an auditory input (e.g., visual /ga/ added on auditory /ba/ leading to the percept of /da/). This led researchers to propose a number of possible architectures for AV integration, according to which auditory and visual information converge toward a single percept in the human brain (Massaro, 1987; Summerfield, 1987; Schwartz et al., 1998).

A number of studies have then searched for potential neurophysiological and neuroanatomical correlates of AV integration in speech perception. At the neurophysiological level, recent electroencephalographic (EEG) and magnetoencephalographic (MEG) studies focused on the influence of the visual input on the auditory event-related potentials (ERPs), notably on auditory N1 (negative peak, occurring typically 100 ms after the sound onset) and P2 (positive peak, occurring typically 200 ms after the sound onset) responses considered to be associated with the processing of the physical and featural attributes of the auditory speech stimulus prior to its categorization (Näätänen and Winkler, 1999). In the last 10 years, various studies consistently displayed an amplitude reduction of N1/P2 auditory responses together with a decrease in their onset latency. These studies typically involved consonant–vowel syllables uttered in isolation, with a natural advance of the visual input (associated with the phonation preparation) on the sound. Their results suggest that the visual input modulates and speeds up the neural processing of auditory ERPs as soon as 100 ms after the sound onset and that AV integration partly occurs at an early processing stage in the cortical auditory speech processing hierarchy (Besle et al., 2004; van Wassenhove et al., 2005; Stekelenburg and Vroomen, 2007; Arnal et al., 2009; Pilling, 2009; Vroomen and Stekelenburg, 2010; Baart et al., 2014; Knowland et al., 2014; Treille et al., 2014a,b). The interpretation has generally called upon “predictive mechanisms” (van Wassenhove et al., 2005), according to which the visual input, arriving ahead of sound, would enable to predict part of its content and hence modulate the auditory ERP in amplitude and latency. The visual modulation seems to obey different rules respectively for N1 and P2. For N1, it would just depend on the advance of the image over the sound, even for incongruent auditory and visual inputs, and even for non-speech stimuli; while the P2 modulation would be speech specific and crucially depend on the phonetic content of the auditory and visual inputs (Stekelenburg and Vroomen, 2007; Vroomen and Stekelenburg, 2010).

While the AV integration process has long been considered as automatic (e.g., Massaro, 1987; Soto-Faraco et al., 2004), a number of recent papers have provided evidence that it could actually be under the control of attentional processes (e.g., Tiippana et al., 2004; Alsius et al., 2005, 2007; Colin et al., 2005; Navarra et al., 2005; Mozolic et al., 2008; Buchan and Munhall, 2012). Furthermore, previous results on the “AV speech detection advantage” (Grant and Seitz, 2000; Kim and Davis, 2004) and its consequences for AV perception (Schwartz et al., 2004) suggest a mechanism by which early visual processing would reduce spectral and temporal uncertainty in the auditory flow. This mechanism, thought to operate prior to AV fusion, would detect whether the visual and acoustic information are bound to the same articulatory event and should be processed together. This view, reinforced by electrophysiological data on early AV speech interactions, suggest that AV interactions could intervene at various stages in the speech decoding process (Bernstein et al., 2004a).

**FIGURE 1:**
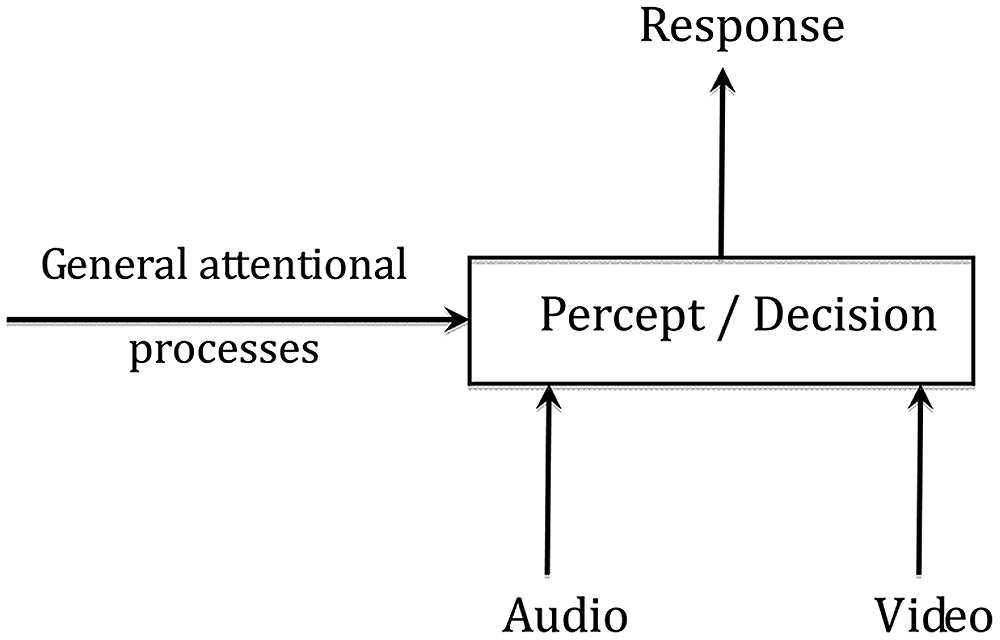
**A two-stage “binding and fusion” model of audiovisual speech perception**.

In a similar vein, Berthommier (2004) proposed that AV fusion could rely on a two-stage process, beginning by binding together the appropriate pieces of auditory and visual information, followed by integration *per se* (**Figure 1**). The binding stage would occur early in the AV speech processing chain enabling the listener to extract and group together the adequate cues in the auditory and visual streams, exploiting coherence in the dynamics of the sound and sight of the speech input. In **Figure 1**, the binding stage is displayed by the output of the “coherence” box assessing the likelihood that the audio and video inputs are indeed associated to the same speech event. The output of the binding stage would provide the input to a second processing stage where categorization (and possibly detection in the AV speech detection paradigm) would occur. Integration would hence occur only at this second stage, and conditioned both by general attentional processes but also by the result of the binding stage. If AV coherence is low, binding is unlikely and integration should be weaker.

**FIGURE 2:**
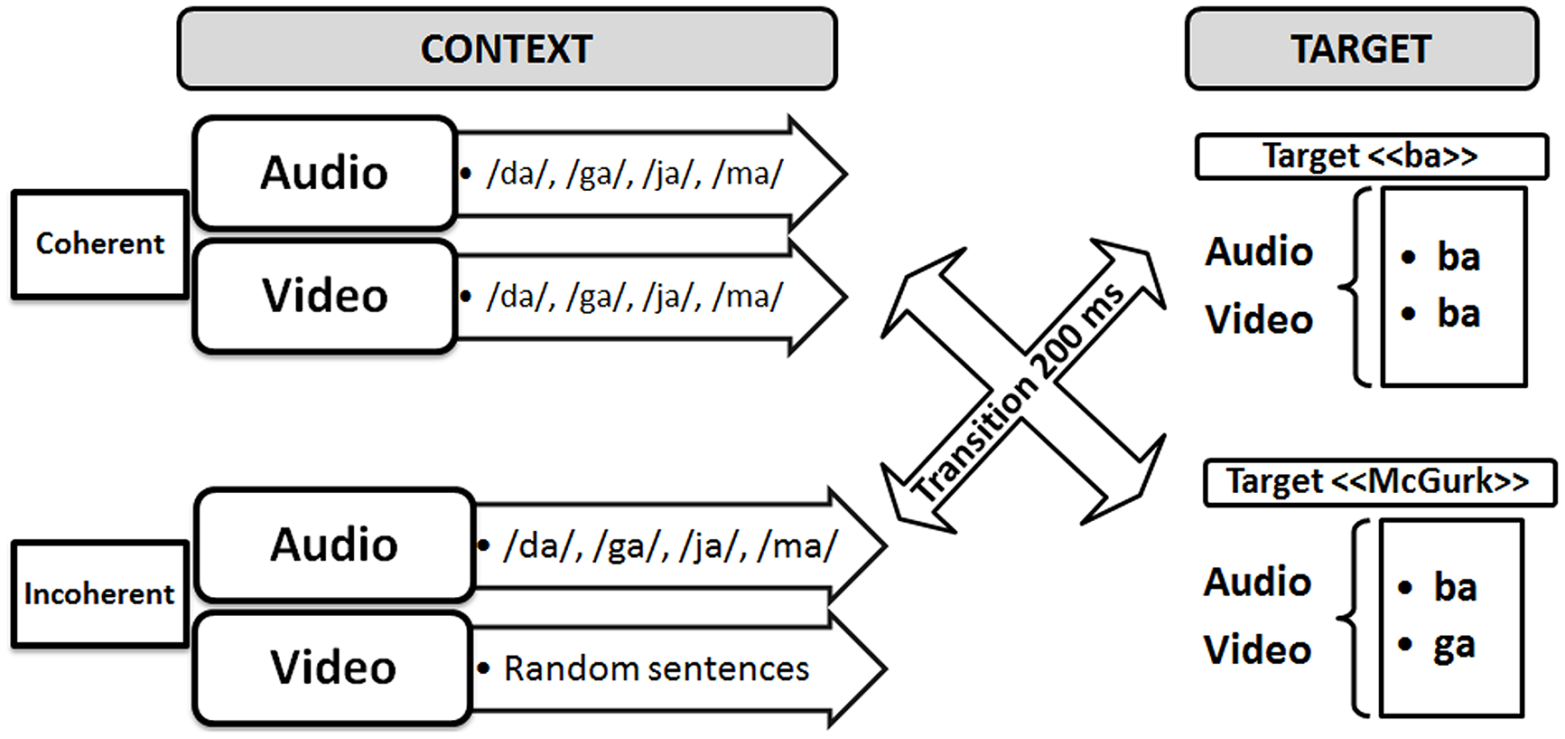
**Experimental paradigm for displaying possible “binding/unbinding” mechanisms where context would modulate the McGurk effect**.

To attempt to demonstrate the existence of this “binding” process, Berthommier and colleagues defined an experimental paradigm possibly leading to “unbinding” (Nahorna et al., 2012). In this paradigm (see **Figure 2**), incongruent “McGurk” (A/ba/ + V/ga/) or congruent “ba” (A/ba/ + V/ba/) targets were preceded by congruent or incongruent AV contexts (to distinguish incongruence in context and in targets, we use the terms “coherent” and “incoherent” for context in the following). The expectation was that the incoherent context should induce the subjects to decrease their confidence that the auditory and visual streams were related to a coherent source. This should decrease the role of the visual input on phonetic decision and hence result in a decrease of the McGurk effect. This is what they called “unbinding.” The experimental results supported this hypothesis. Indeed, compared to the coherent contexts, various kinds of incoherent contexts, such as acoustic syllables dubbed on video sentences, or phonetic or temporal modifications of the acoustic content of a regular sequence of AV syllables, produced significant amounts of reduction in the McGurk effect. In line with the two-stage model of AV fusion (see **Figure 1**), these results suggest that fusion can be conditioned by prior contexts on AV coherence. They also appear compatible with the above-cited behavioral data on AV detection suggesting that the coherence of the auditory and visual inputs is computed early enough to enhance auditory processing, resulting in the AV speech detection advantage.

The present study aimed at determining a possible neurophysiological marker of the AV binding/unbinding process in the cortical auditory speech hierarchy. Capitalizing on the results obtained by Nahorna et al. (2013), the experiment was adapted from previous EEG experiments on AV speech perception, adding either a coherent or an incoherent AV context before auditory, congruent AV and incongruent AV speech stimuli. The assumption is that with coherent context we should replicate the results of previous EEG studies on auditory N1/P2 responses (decrease in amplitude and latency in the AV vs. A condition). However, an incoherent context should lead to unbinding, as in Nahorna et al. (2013), with the consequence that the visual influence on the auditory stimulus should decrease. Hence the N1/P2 latency and amplitude in the AV condition should increase (reaching a value close to their value in the A condition) in the incoherent context compared with the coherent context.

## MATERIALS AND METHODS

### PARTICIPANTS

Nineteen healthy volunteers (17 women and 2 men, mean age = 30 years, SD = 13.1 years) participated in the experiment. All participants were French native speakers (although no standard tests were used to measure first or, possibly, second language proficiency), right-handed, without any reported history of hearing disorders and with normal or corrected-to-normal vision. Written consent was obtained from each participant and all procedures were approved by the Grenoble Ethics Board (CERNI). The participants were paid for participating in the experiment.

### STIMULI

**FIGURE 3:**
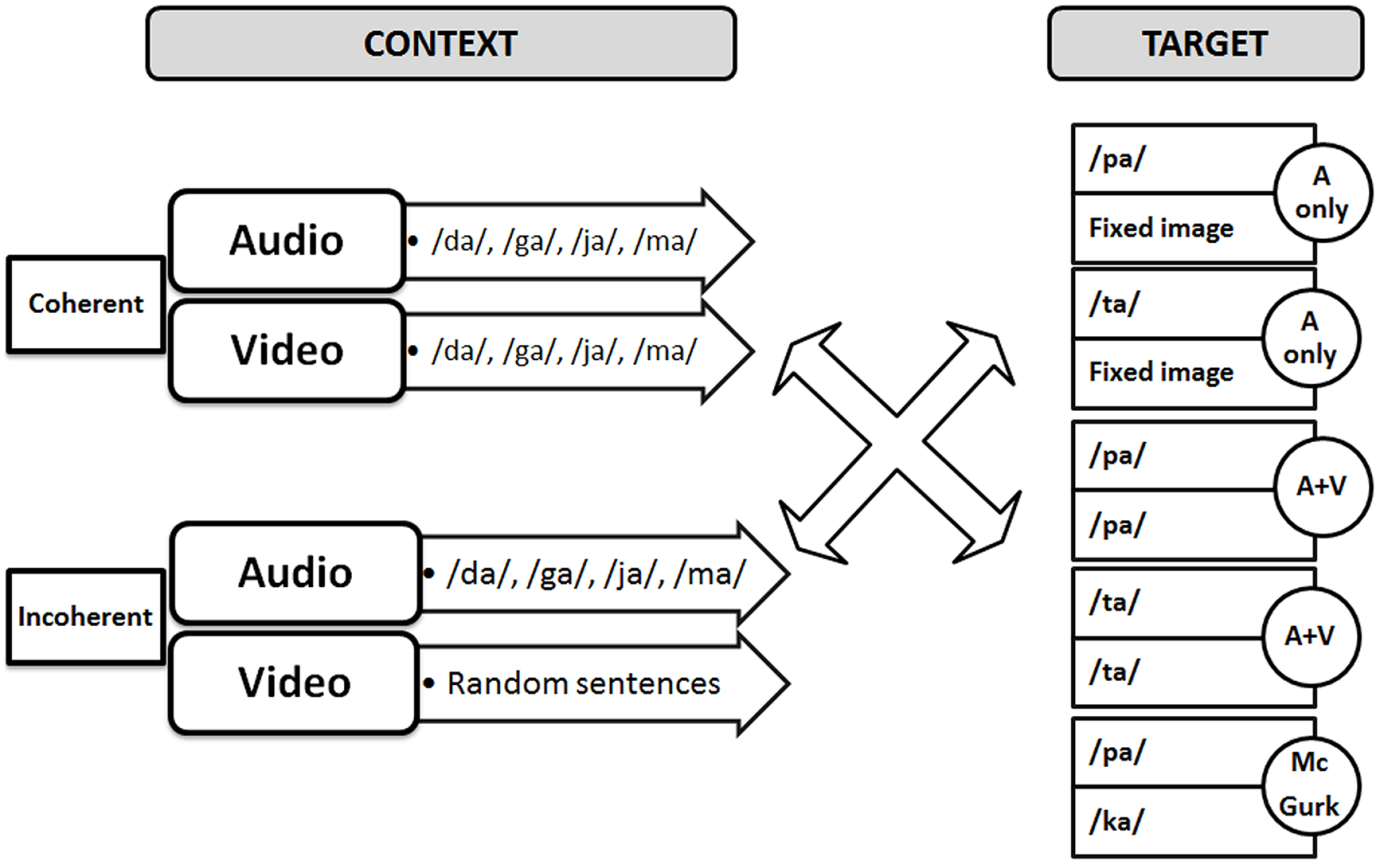
**Audiovisual material used in the experiment**.

The audio–video stimuli were similar to those of the previous experiments by Nahorna et al. (2012, 2013) that is with an initial part called “context” followed by a second part called “target.” The target was either a pure audio stimulus (“pa” or “ta” dubbed with a fixed image for the same duration), or a congruent AV stimulus (“pa” or “ta”) or an incongruent “McGurk” stimulus (audio “pa” dubbed on a video “ka”). The AV context was either coherent or incoherent (**Figure 3**). Coherent contexts consisted of regular sequences of coherent AV syllables randomly selected within the following syllables (“va,” “fa,” “za,” “sa,” “ra,” “la,” “ja,” “cha,” “ma,” “na”). These syllables were selected within the set of possible /Ca/ syllables in French, where C is a consonant not contained in the /p t k b d g/ set, so that target syllables /pa ta ka/ or their perceptually close voiced counterparts /ba da ga/, cannot appear in the context. In the incoherent context material, the auditory content was the same, but the visual content was replaced by excerpts of video sentences, produced in a free way by the same speaker, and matched in duration. The context and target, both of fixed duration (respectively 2 and 1.08 s), were separated by a 1 s period of silence and fixed black image.

All stimuli were prepared from two sets of AV material, a “syllable” material and a “sentence” material, produced by a French male speaker, with lips painted in blue to allow precise video analysis of lip movements (Lallouache, 1990). Videos were edited in Adobe Premier Pro into a 720/576 pixel movie with a digitization rate of 25 frames/s (1 frame = 40 ms). Stereo soundtracks were digitized in Adobe Audition at 44.1 kHz with 16-bit resolution.

**FIGURE 4:**
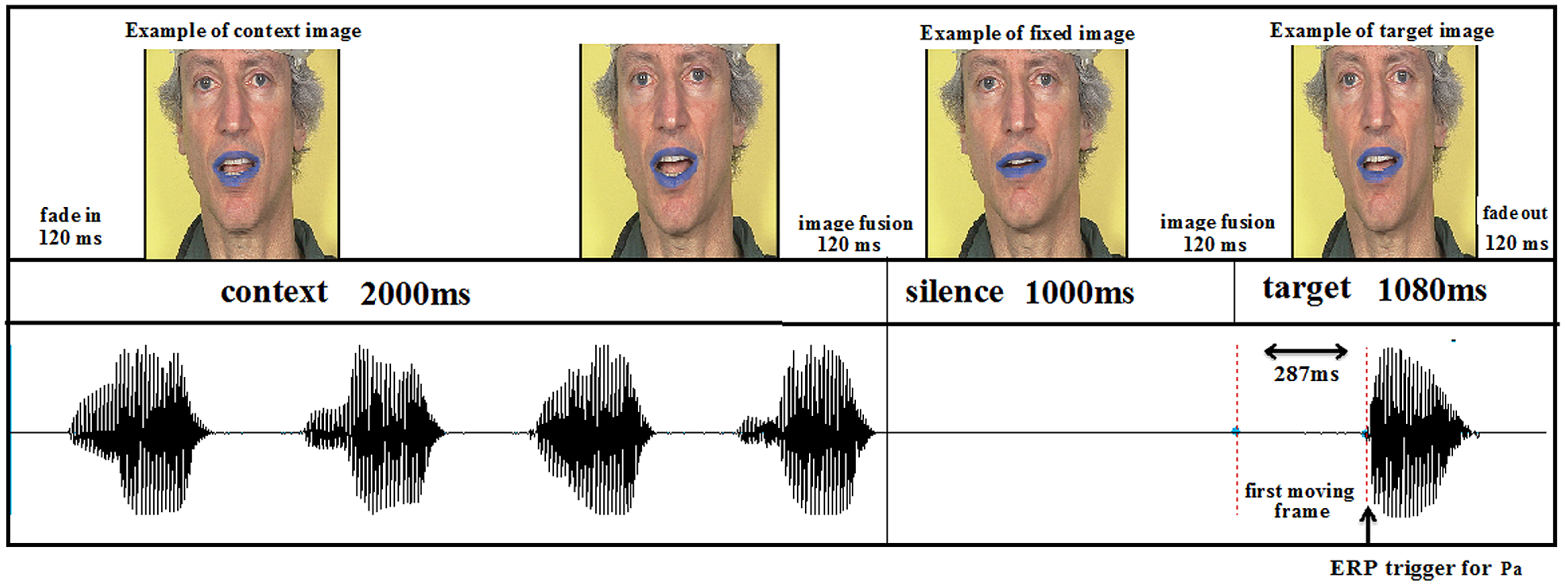
**Experimental sequence**.

The duration of each trial was 5280 ms, in which the context AV movie, lasting 2000 ms, was followed by silence for 1000 ms, then by the target with a duration of 1080 ms. The response time was 1200 ms. To ensure continuity between the end of the context stimulus and silence and also between silence and the onset of the target stimulus, a 120-ms transition stimulus was included by image fusion (see **Figure 4**). Video fade-in and fade-out were also included in the first and last three frames, respectively. In the auditory only conditions, the auditory targets were presented with a static face of the speaker. The difference between the visual and auditory onsets for /pa/ and /ta/ were respectively 287 and 206 ms.

### PROCEDURE

The subject’s task was to categorize the stimuli as “pa” or “ta,” by pressing the appropriate key (two-alternative forced-choice identification task). Stimulus presentation was coordinated with the Presentation software (Neurobehavioral Systems). In order to avoid possible interference between speech identification and motor response induced by key pressing participants were told to produce their responses a short delay after the stimulus end when a question mark symbol appeared on the screen (typically 320 ms after the end of the stimulus). There were six conditions, with three targets (audio-only, A vs. AV congruent, AVC vs. AV incongruent, AVI) and two contexts (coherent vs. incoherent), and altogether 100 repetitions per condition (with 50 “pa” and 50 “ta” in the audio-only or AV congruent targets, and 100 McGurk stimuli). This provided altogether 600 occurrences, presented in a random order inside five experimental blocks. Altogether, the experiment lasted more than 1 h, including subject preparation, explanations and pauses between blocks. This unfortunately removed the possibility to add a specific visual-only condition, since it would have added two targets – visual congruent and visual incongruent – and hence almost doubled the experiment duration. We will discuss in various parts of the paper what the consequences of this specific choice could be in the processing and interpretation of EEG data.

The experiment was carried out in a soundproof booth with the sound presented through a loudspeaker at a comfortable and fixed level for all subjects. The video stream was displayed on a screen at a rate of 25 images per second, the subject being positioned at about 50 cm from the screen. Participants were instructed to categorize each target syllable by pressing on one key corresponding to /pa/ or /ta/ on a computer keyboard (with a counterbalanced order between subjects) with their left hand.

### EEG PARAMETERS

Electroencephalography data were continuously recorded from 64 scalp electrodes (Electro-Cap International, Inc., according to the international 10–20 system) using the Biosemi ActiveTwo AD-box EEG system operating at a 256 Hz sampling rate. Two additional electrodes served as reference [common mode sense (CMS) active electrode] and ground [driven right leg (DRL) passive electrode]. One other external reference electrode was put at the top of the nose. Electro-oculogram measures of the horizontal (HEOG) and vertical (VEOG) eye movements were recorded using electrodes at the outer canthus of each eye as well as above and below the right eye. Before the experiment, the impedance of all electrodes was adjusted to get low offset voltages and stable DC.

### ANALYSES

All EEG data were processed using the EEGLAB toolbox (Delorme and Makeig, 2004) implemented in Matlab (Mathworks, Natick, MA, USA). EEG data were first re-referenced off-line to the nose recording and band-pass filtered using a two-way least-squares FIR filtering (2–20 Hz). Data were then segmented into epochs of 600 ms including a 100 ms pre stimulus baseline, from –100 to 0 ms to the acoustic target syllable onset, individually determined for each stimulus from prior acoustical analyses). Epochs with an amplitude change exceeding ±100 μV at any channel (including HEOG and VEOG channels) were rejected (<5%).

As previously noted, because of time limitations a visual-alone condition was not incorporated in the study, while it is generally included in EEG studies on AV perception. However, to attempt to rule out the possibility that visual responses from the occiptal areas could blur and contaminate auditory evoked responses in fronto-central electrodes, we performed various topography analyses using EEGLAB to define the spatial distributions and dynamics of the activity on the scalp surface. Fp1, Fz, F2, P10, P9, and Iz electrodes were not included in this analysis because of noisy electrodes or dysfunction of electrodes for at least one participant. We studied the spatial distribution in two steps. Firstly, we plotted the scalp maps for all six conditions (context × modality) to confirm that the maximal N1/P2 auditory evoked potentials were indeed localized around fronto-central sites on the scalp. The aim of the second step was to evaluate the presence and amount of possible contamination in the auditory fronto-central electrodes by the visual responses in corresponding cortical areas dedicated to the processing of visual information. To do so, we calculated scalp maps between conditions in the N1-P2 time period.

**FIGURE 5:**
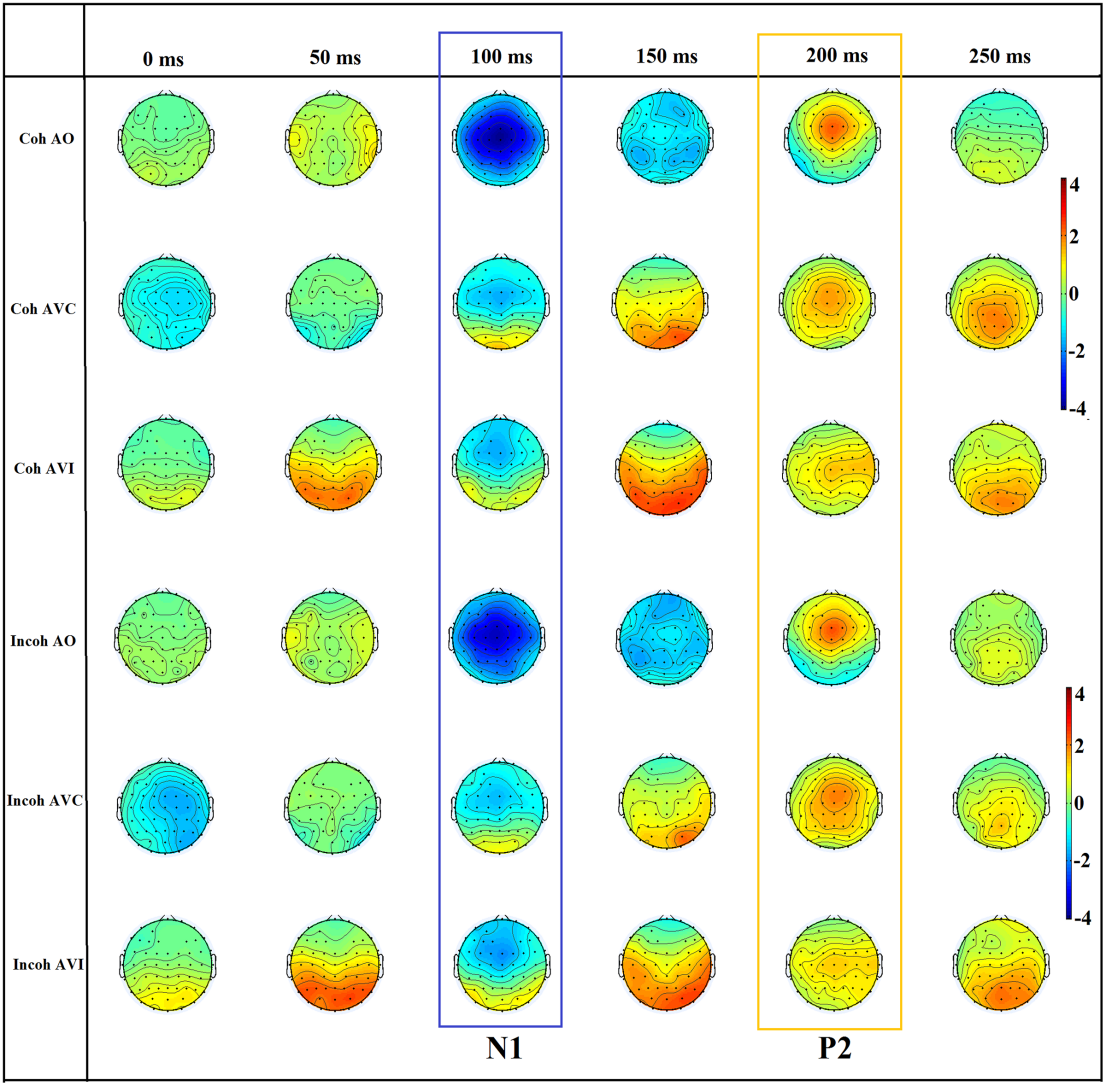
**The scalp topography of N1 and P2 for the six conditions (Coh AO, Coh AVC, Coh AVI, Incoh AO, Incoh AVC, Incoh AVI) in time steps of 50 ms.** The range of the voltage maps is from –4 to 4 μv.

Since the first part of the topographic analysis confirmed that maximal N1/P2 auditory evoked potentials indeed occurred over fronto-central sites on the scalp (see **Figure 5**; see also Scherg and Von Cramon, 1986; Näätänen and Picton, 1987), and in line with previous EEG studies on AV speech perception and auditory evoked potentials (e.g., van Wassenhove et al., 2005; Stekelenburg and Vroomen, 2007; Pilling, 2009; Vroomen and Stekelenburg, 2010; Treille et al., 2014a,b), an ERP analysis was then conducted on six representative left, middle, and right fronto-central electrodes (F3, Fz, F4, C3, Cz, C4) in which AV speech integration has been previously shown to occur (note that Fz was replaced by the average of F1 and F2 responses for two participants because of a dysfunction of electrodes). For each participant, the peak latencies of auditory N1 and P2 evoked responses were first manually determined on the EEG waveform averaged over all six electrodes for each context and modality. Two temporal windows were then defined on these peaks ±30 ms in order to individually calculate N1 and P2 amplitude and latency for all modalities, context and electrodes. Peak detection was done automatically.

For P2 amplitude and latency it has to be noticed that the N1-to-P2 latency could reach small values as low as 75 ms, with double P2 peaks for many subjects. This is not unclassical: double peaks in the P2 time period have actually been found in a number of studies in both adults, children, elderly and also in impaired populations (e.g., Ponton et al., 1996; Hyde, 1997; Ceponiene et al., 2008; Bertoli et al., 2011). Since the classical range for P2 is 150–250 ms and since the first P2 peak was close to this range, the analysis was focused on the first P2 peak for further analyses.

Notice that we also tested another baseline earlier on in the silence portion between context and target that is from –500 to –400 ms to the acoustic target syllable onset, and we checked that this did not change the results presented later, in any crucial way, either in whole graphs or statistical analysis.

Repeated-measure analyses of variances (ANOVAs) were performed on N1 and P2 amplitude and latency with context (coherent vs. incoherent) and modality (A vs. AVC vs. AVI) as within-subjects variables. Partial eta squared values were systematically provided to estimate effect sizes. *Post hoc* analyses with Bonferroni correction were done when appropriate, and are reported at the *p* < 0.05 level.

Concerning behavioral data, the proportion of responses coherent with the auditory input was individually determined for each participant, each syllable, and each modality. A repeated-measure ANOVA was performed on this proportion with context (coherent vs. incoherent) and modality (A vs. AVC vs. AVI) as within-subjects variables. *Post hoc* analyses with Bonferroni correction were done when appropriate, and are reported at the *p* < 0.05 level.

## RESULTS

### BEHAVIORAL ANALYSIS

**FIGURE 6:**
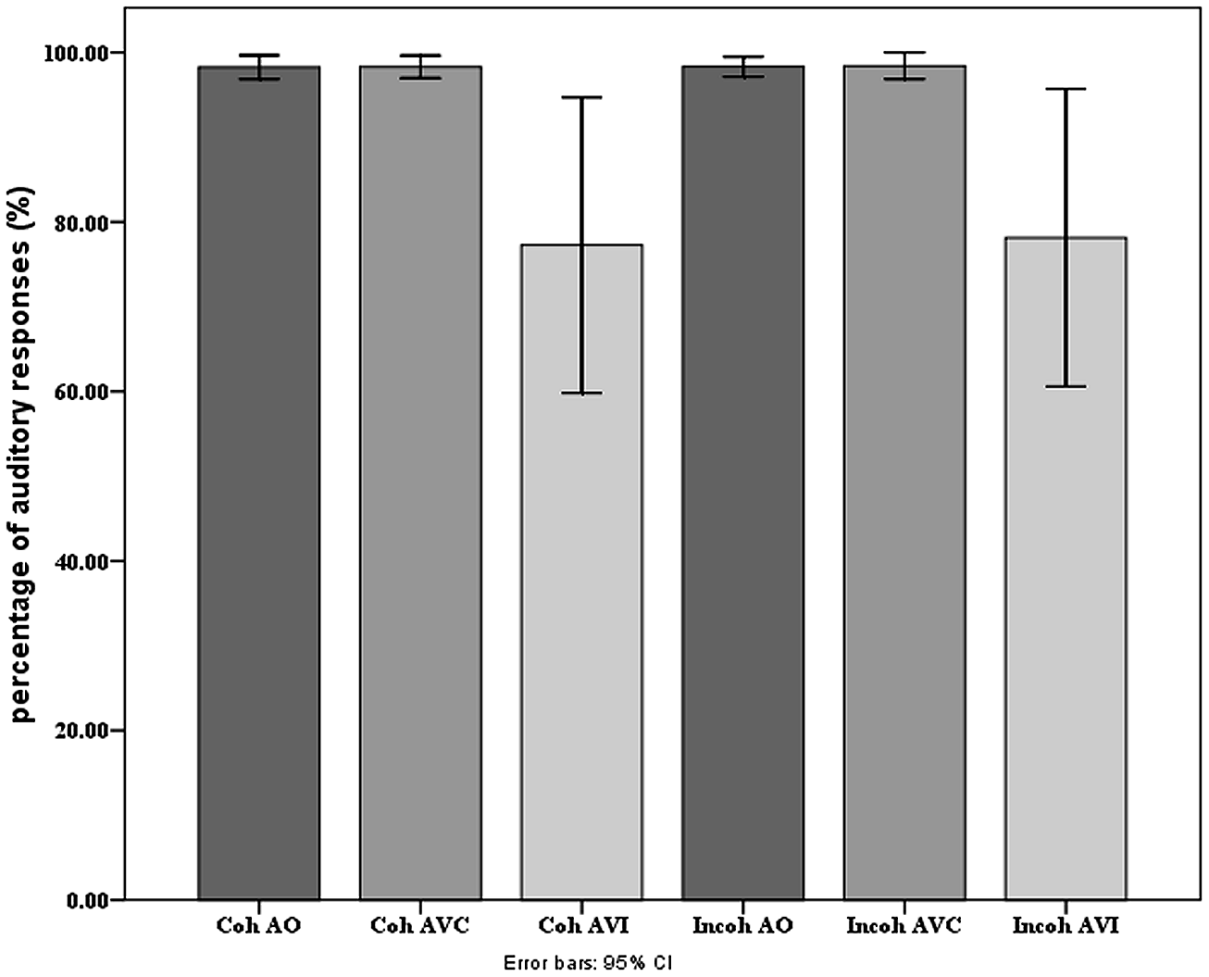
**Mean percentage of responses coherent with the auditory input in each modality and context presentation in the behavioral experiment.** Error bars represent standard errors of the mean.

On **Figure 6** we display the behavioral scores, presented as percentage of responses coherent with the auditory input. The scores were close to 100% in the A and AV conditions. They were lower in the AVI conditions, since the visual input changes the percept and produces some McGurk effect. The main effect of modality of presentation was significant [*F*(2,36) = 6.14, *p* < 0.0.05], with more correct responses in A and AVC than in AVI modalities (as shown by *post hoc* analyses; on average, A: 98.2%, AV: 98.3%, and AVI: 77.7%). There was no significant effect of context or interaction. Contrary to our previous studies (Nahorna et al., 2012, 2013), the amount of McGurk effect is hence very small and independent on context. This is likely due to the specific procedure associated with EEG experiments in which the number of different stimuli is quite low (only five different target stimuli altogether) with highly predictable targets.

### EEG ANALYSES

#### N1 amplitude and latency (see **Figures 7** and **8A,B**)

In the following analysis, N1 amplitudes were reported in absolute values, hence reduced amplitude means a reduction in absolute value and an increase in real (negative) values. The repeated-measures ANOVA on N1 amplitude displayed no significant effect of context, but a significant effect of modality [*F*(2,36) = 13.29, *p* < 0.001, $\eta_{p}^{2}$ = 0.42], with a reduced N1 amplitude observed for the AVC and AVI modalities as compared to the A modality (**Figure 8A**). The *post hoc* analysis shows that the amplitudes in both AVC (–2.00 μV) and AVI (–1.64 μV) were indeed smaller compared to A (–3.62 μV) irrespective of context. Interaction between context and modality was not significant.

**FIGURE 7:**
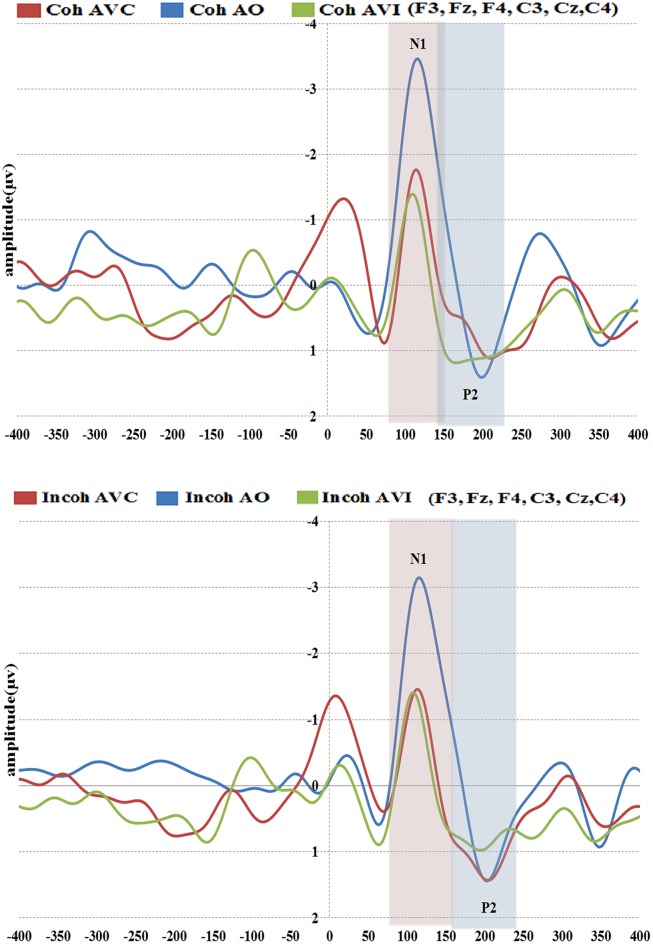
**Grand-average of auditory evoked potentials for the six electrodes (frontal and central) for coherent (top) and incoherent (bottom) context and in the three conditions (AO, AVC, and AVI)**.

**FIGURE 8:**
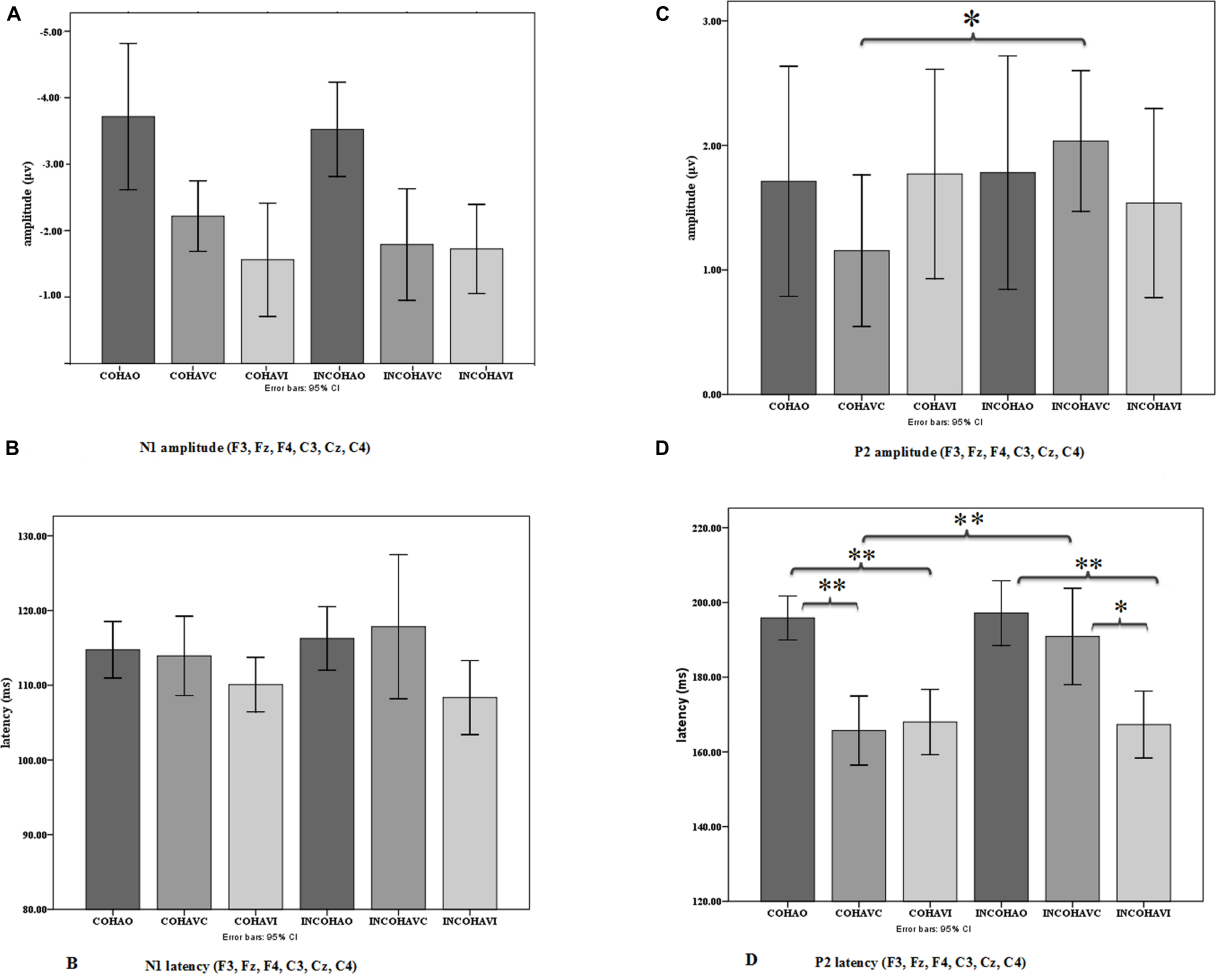
**Mean N1 **(A)** and P2 **(C)** amplitude and mean N1 **(B)** and P2 **(D)** latency averaged over the six electrodes in the three conditions (AO, AVC, and AVI) and the two contexts (coherent and incoherent).** Error bars represent standard errors of the mean. *p = 0.05; **p = 0.005.

The repeated-measures ANOVA on N1 latency displayed no significant effect of context (**Figure 8B**). The modality effect was close to significance [*F*(2,36) = 3.20, *p* = 0.07, $\eta_{p}^{2}$ = 0.15], with a shorter latency in the AVI (109 ms) compared to the A (115 ms) and AVC (115 ms) conditions. Interaction between context and modality was not significant.

In brief the results about N1 amplitude are similar to the previously mentioned EEG studies on AV speech perception, with a visually induced amplitude reduction for both congruent (AVC) and incongruent (AVI) stimuli irrespective of context. Regarding N1 latency, the difference between auditory and AV modalities is smaller than in few previous EEG studies, and consequently not significant.

#### P2 amplitude and latency (see **Figures 7** and **8C,D**)

There was no significant effect of context or modality in P2 amplitude, but the interaction between context and modality was significant [*F*(2,36) = 3.51, *p* < 0.05, $\eta_{p}^{2}$ = 0.16], which is in line with our hypothesis (**Figure 8C**). To further examine the interaction effect between context and modality in P2 amplitude, pairwise comparisons were done using Bonferroni corrections to test the effect of context separately for each modality. The *post hoc* analysis within modality provided a significant difference between Coherent and Incoherent AVC conditions (*p* = 0.01), showing that Coherent AVC (1.15 μV) has smaller amplitude compared to Incoherent AVC (2.03 μV). Context provided no other significant differences either in the AVI or in the A modality.

Concerning P2 latency (**Figure 8D**), there was a significant effect of context [*F*(1,18) = 5.63, *p* < 0.05, $\eta_{p}^{2}$ = 0.23], the latency in the Coherent context (176 ms) being smaller than in the Incoherent context (185 ms). There was also a significant effect of modality [*F*(2,36) = 23.35, *p* < 0.001, $\eta_{p}^{2}$ = 0.56], P2 occurring earlier in the AVC (178 ms) and AVI (167 ms) modalities compared to AO (196 ms). As in the case of P2 amplitude, there was a significant interaction effect between context and modality [*F*(2,36) = 8.07, *p* < 0.005, $\eta_{p}^{2}$ = 0.31]. The *post hoc* analysis provided a significant difference between Coherent and Incoherent AVC conditions (*p* = 0.002), showing that P2 in the Coherent AVC condition occurred earlier (165 ms) than in the Incoherent AVC condition (190 ms). Context provided no other significant differences either in the AVI or in the A modality.

Therefore, contrary to the data for N1, we observed significant effects of context for P2. These effects concern both amplitude and latency. They are focused on the AVC condition with rather large values (25 ms increase in latency and 0.88 μV increase in amplitude from Coherent to Incoherent context in the AVC condition). They result in removing the latency difference between AVC and A, in line with our expectations. However, there appears to be no effect of context in the AVI condition, neither for amplitude nor for latency.

#### Scalp topographies and the potential role of a contamination from visual areas (see **Figures 9A–D**)

**FIGURE 9:**
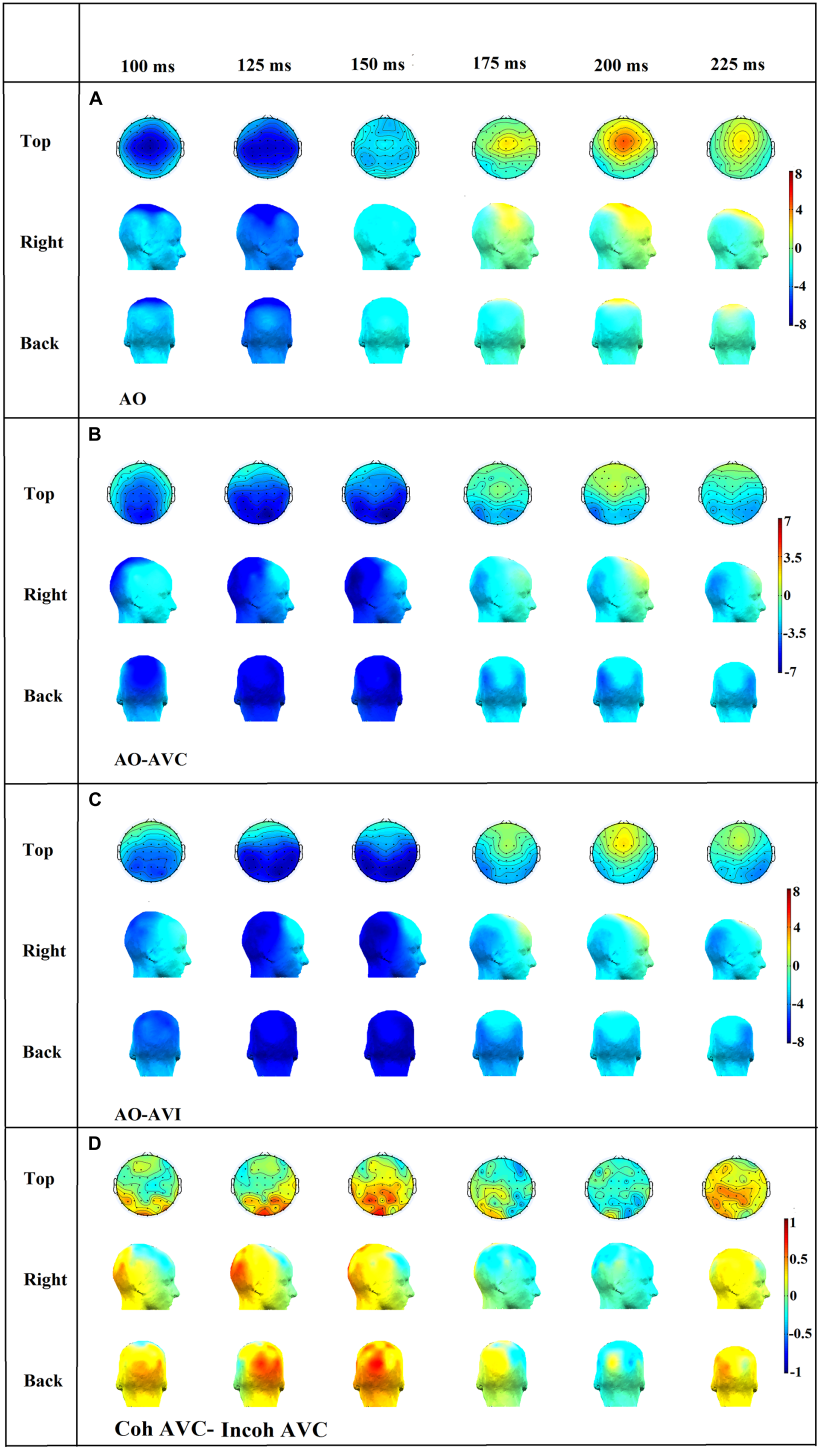
**Topographical distributions of the grand average ERPs for the AO **(A)**, AO-AVC **(B)**, AO-AVI **(C)** and Coh AVC-Incoh AVC **(D)** different waves in time steps of 25 ms.** The range of the voltage maps varies between maps, but is always expressed in μv.

To assess potential contamination of the previous responses by visually driven responses from the visual cortex, we analyzed scalp topographies in the N1-P2 time periods in various conditions. Firstly we assessed whether visual areas could intervene in the visual modulation of N1 and P2 responses in the congruent and incongruent configurations, independently on context, by comparing the AO condition (**Figure 9A**) with either the AVC (**Figure 9B**) or the AVI (**Figure 9C**) condition (averaging responses over context, that is combining Coherent AVC and Incoherent AVC in **Figure 9B** and Coherent AVI and Incoherent AVI in **Figure 9C**).

In the N1 time period (100–150 ms) it appeared that the negative peak value was more prominent in central than in occipital electrodes (**Figure 9A**), but the decrease in N1 amplitude in central electrodes in both AVC and AVI conditions, associated with a negative amplitude in central electrodes in both AO-AVC and AO-AVI maps (**Figures 9B,C**) was accompanied by an even larger negative amplitude in occipital electrodes. This is due to a positive peak in AV conditions corresponding to the arrival of the visual response in this region. Therefore a possible contamination of the visual influence on N1 response due to occipital activity cannot be discarded at this stage.

In the P2 time period (175–225 ms), once again the positive peak was more prominent in central than in occipital electrodes (**Figure 9A**). The AO-AVC and AO-AVI scalp maps (**Figures 9B,C**) displayed positive values in central electrodes, corresponding to a decrease in P2 amplitude from AO to both AV conditions. Contrary to what happened for N1, the situation in occipital electrodes was here completely reversed: there were indeed negative values of AO-AVC and AO-AVI differences in the occipital region. Therefore, the possible contamination of visual effects on P2 by visual responses is much less likely than for N1.

Finally, to directly assess possible contaminations on the major effect of interest that is the difference between incoherent and coherent contexts in the AVC condition, we computed scalp topographies for the difference between coherent AVC and incoherent AVI conditions (see **Figure 9D**). The differences were rather small all over these maps, and the topography differences were globally relatively noisy and make difficult any clear-cut conclusion from these topography.

Altogether, the results in the coherent context condition seem partially consistent with previous findings of EEG studies, if we assume that the Coherent context provides a condition similar to previous studies with no-context. Visual speech in the congruent AVC and incongruent AVI conditions is associated to both a significant decrease in amplitude for N1 and in latency for P2. Importantly we found a significant effect of context in the AVC condition for both amplitude and latency in P2, in line with our prediction. However, scalp topographies raise a number of questions and doubts on the possibility to unambiguously interpret these data, in the absence of a visual-only condition. We will now discuss these results in relation with both previous EEG studies on AV speech perception and with our own assumptions on AV binding.

## DISCUSSION

Before discussing these results it is necessary to consider one important potential limitation of the present findings. Testing cross-modal interactions usually involves determining whether the observed response in the bimodal condition differs from the sum of those observed in the unimodal conditions (e.g., AV ≠ A + V). In the present study, as previously noted, the visual-alone condition was not obtained because of time limitation. Although direct comparison between AV and auditory conditions performed in previous EEG studies on AV speech integration have provided fully coherent results with other studies using an additive model (see van Wassenhove et al., 2005; Pilling, 2009; Treille et al., 2014a,b), this limitation is important, and will lead to a specific component of our discussion.

### COMPARISON OF THE COHERENT CONTEXT CONDITIONS WITH PREVIOUS EEG STUDIES

A preliminary objective of the study was to replicate the results of previous EEG studies on N1/P2 in coherent context (Klucharev et al., 2003; Besle et al., 2004; van Wassenhove et al., 2005; Stekelenburg and Vroomen, 2007, 2012; Pilling, 2009; Vroomen and Stekelenburg, 2010; Baart et al., 2014; Knowland et al., 2014; Treille et al., 2014a,b). Concerning AV congruent stimuli AVC, our data are partially in line with previous studies. For the N1 component, we obtained an amplitude reduction in AVC compared to AO, as in previous studies (**Figure 8A**), though this amplitude reduction was not accompanied by a latency reduction (**Figure 8B**), contrary to previous studies. In the P2 component, the decrease in amplitude and latency (**Figures 8C,D**) from AO to AVC is also in line with previous studies (e.g., van Wassenhove et al., 2005; Stekelenburg and Vroomen, 2007; Pilling, 2009; Vroomen and Stekelenburg, 2010; Knowland et al., 2014). Concerning AV incongruent (“McGurk”) stimuli AVI, there was an amplitude reduction compared to the AO condition for N1 (**Figure 8A**) and the two peaks also occurred earlier than in the AO condition, not significantly in N1 (**Figure 8B**) but significantly in P2 (**Figure 8D**). Here, the output of previous studies is more contrasted. As a matter of fact, the N1 amplitude and latency values for incongruent stimuli are not available in the van Wassenhove et al. (2005) study, whereas in the studies by Stekelenburg and Vroomen (2007) and Baart et al. (2014) there is no difference between incongruent and congruent conditions on both amplitude and latency. However, the results for P2 are not consistent with the previous studies that compared congruent and incongruent stimuli, e.g., in the study by Stekelenburg and Vroomen (2007) there is an effect of incongruent stimuli on amplitude but no effect on latency whereas in the study by van Wassenhove et al. (2005) there is no amplitude effect but a latency effect. On the contrary, the recent study by Knowland et al. (2014) is in line with the present findings in the incongruent condition for N1 and P2 amplitude, even though the stimulus for incongruency differs from the present study. Of course, some of these differences could also be due to various methodological differences in the analyses, including in the present case the specific choice to systematically keep the first peak in the P2 region in the case of double peaks responses, which occur for many subjects (see Analyses).

### COMPARISON OF THE COHERENT AND INCOHERENT CONTEXT CONDITIONS IN THE PRESENT STUDY

The primary objective of the study was to test the possible role of an incoherent context supposed to lead to unbinding (as robustly displayed by behavioral data in Nahorna et al., 2012, 2013) and hence decrease the effects of the visual input on N1/P2 latency and amplitude.

We obtained no effect of context, either alone or in interaction with modality, for both N1 amplitude and latency (**Figures 8A,B**). However, we obtained a significant effect of context for P2, alone for latency, and in interaction with modality for both latency and amplitude. *Post hoc* tests showed that these effects could be due to a suppression of the decrease in amplitude and latency from AO to AVC when the context is incoherent (**Figures 8C,D**).

The fact that there is an effect of context for P2 but not for N1 is coherent with the view that these components could reflect different processing stages, AV effects on N1 possibly being not speech specific and only driven by visual anticipation independently on AV phonetic congruence, while P2 would be speech specific, content dependent and modulated by AV coherence (Stekelenburg and Vroomen, 2007; Baart et al., 2014). In summary, the visual modality would produce a decrease in N1 amplitude and possibly latency because of visual anticipation, independently on target congruence and context coherence. A congruent visual input (AVC) would lead to a decrease in P2 amplitude and latency in the coherent context because of visual predictability and AV speech-specific binding. This effect would be suppressed by incoherent context because of unbinding due to incoherence.

As for AVI stimuli, there was no context effect, both in behavioral and EEG results. Actually, it appears that there is almost no AV integration in the present study for incongruent McGurk stimuli (as shown by behavioral data), which likely explains the lack of a role of context on EEG for these stimuli. The discrepancy in behavioral data with previous experiments by Nahorna et al. (2012, 2013) likely comes from differences in the nature and number of stimuli. The studies by Nahorna et al. (2012, 2013) involved voiced stimuli “ba,” “da,” and “ga” whereas in the present study the EEG requirement to avoid prevoicing, classical in the French language, forced us to select unvoiced stimuli “pa,” “ta,” and “ka.” More importantly, the previous studies were based on a larger level of unpredictability, the subjects did not know when the targets would happen in the films, and the coherent and incoherent contexts were systematically mixed. In the present study, because of the constraints in the EEG paradigm, there were no temporal uncertainty of the time when the target occurred, and the AV material was highly restricted, with only 10 different stimuli altogether (five different targets and two different contexts). A perspective would hence be to use more variable stimuli in a further experiment.

The difference between AO and AVI conditions in P2 latency and amplitude could be related to the fact that the subjects detect an AV incongruence. Indeed, behavioral data in Nahorna et al. (2012, 2013) consistently display an increase in response times for McGurk stimuli compared with congruent stimuli, independently on context, and this was interpreted by the authors as suggesting that subjects detected the local incongruence independently on binding *per se*, while binding would modulate the final decision. In summary, AVI would produce (i) decrease in N1 amplitude and possibly latency because of visual anticipation; (ii) decrease of P2 amplitude and latency because of incongruence detection; (iii) but no integration *per se*, as displayed by behavioral data, and hence no modulation by context and binding/unbinding mechanisms.

At this stage, and keeping for a while this global interpretation compatible with the “binding” hypothesis, it is possible to come back to the two-stage AV fusion process (**Figure 1**). The present EEG data add some information about the way coherence could be computed for congruent stimuli. If indeed the P2 AV modulation in amplitude and latency is related to the binding mechanism as supposed by, e.g., Baart et al. (2014), then the evaluation of coherence, supposed by Nahorna et al. (2012, 2013) to take place in the context period before the target, should apply for both congruent and incongruent stimuli. Actually, modulation of binding by context has been shown in behavioral data on incongruent stimuli in previous studies, and in P2 data on congruent stimuli in the present study. Altogether, this suggests that the two-stage process described in **Figure 1** could operate, at least in part, prior to P2. These findings will have to be confirmed by future EEG experiments on more variable stimuli able to provide P2 modulation for both congruent and incongruent stimuli, and possibly in other kinds of attentional processes.

### POSSIBLE CONTAMINATION BY VISUAL AREAS AND SUGGESTIONS FOR FUTURE STUDIES

A crucial limitation of the present work is the lack of a visual-only condition. We consider that this was a necessary evil in such a preliminary study, since it was the only way to be able to assess both congruent and incongruent targets in coherent vs. incoherent contexts. But this might have resulted in possible contamination effects from visual regions that we will discuss now.

Firstly, contamination could be due to visual context. This is, however, rather unlikely considering that the different contexts finish 1000 ms before the target. We systematically compared results obtained with two baseline conditions, one far from the end of the context (–100 to 0 ms) and the other one closer (–500 to –400 ms). It appeared that this baseline change did not change the current results in any crucial way, either in whole graphs or statistical analysis, which suggests that the fluctuations in ERP responses before the apparition of the auditory stimulus at 0 ms do not intervene much in the further analysis of AV interactions on N1 and P2.

It is more likely that contamination effects could be due to visual responses to the visual component of the target. This appears particularly likely in the N1 time period, where scalp maps in the AO-AVC and AO-AVI conditions (**Figure 9**) display larger negative values in occipital areas than in central electrodes. Therefore, it cannot be ruled out that (some unknown) part of the visual modulation of the auditory response could be due to propagation of visual responses from the occipital region.

In the P2 time period this is much less likely, considering that the pattern of responses is now completely inverse between central and occipital electrodes, with a decrease of P2 amplitude from AO to AVC or AVI in the first ones, and an increase in the second ones. However, the pattern of scalp difference between coherent and incoherent AVC conditions is complex and fuzzy, and the amplitude differences between conditions are small. Therefore, we cannot discard the possibility that the modulation of P2 response in the incoherent compared with coherent context is due to propagation of the visual activity – though we must remind that in these two conditions, the visual response actually corresponds to exactly the same visual input, which makes the “visual propagation” hypothesis more unlikely.

Altogether our interpretation of the observed results is that (1) the pattern of EEG responses we obtained in the N1-P2 time periods is compatible with classical visual effects on the auditory response in this pattern of time, and with a possible modulation of these effects by AV context, in line with our assumptions on AV binding; (2) however, the lack of a visual-only condition impedes to firmly discard other interpretations considering contamination from visual regions due to responses to the visual component of the stimulus; and (3) this suggests that more experiments using the same kind of paradigm with AV context, incorporating visual-only conditions to enable better control of the visual effects are needed to assess the possibility to exhibit electrophysiological correlates of the binding/unbinding mechanism in the human brain.

## CONCLUSION

We displayed a new paradigm for ERP AV studies based on the role of context. We presented data about modulation of the auditory response in the N1-P2 time periods due to the visual input, both in the target and context portions of the stimulus. We proposed a possible interpretation of the modulations of the N1 and P2 components, associated to (1) a classical visual modulation generally associated with predictive mechanisms (see e.g., van Wassenhove et al., 2005) and (2) possible modifications of this effect due to incoherent context, in the framework of the two-stage “binding and fusion” model proposed by Nahorna et al. (2012). However, we also discussed in detail a concurrent interpretation only based on the contamination by visual responses in the visual regions, due to the impossibility in the present study to incorporate a visual-only condition.

The search for electrophysiological correlates of attentional processes possibly modifying AV interactions is an important challenge for research on AV speech perception (see e.g., the recent study by Alsius et al. (2014) measuring the effect of attentional load on AV speech perception using N1 and P2 responses as cues just as in the present study). We suggest that binding associated with context should be integrated in general descriptions of AV modulations of the N1 and P2 components of auditory ERP responses to speech stimuli, in relation with general and speech specific effects and the role of attention.

## Conflict of Interest Statement

The authors declare that the research was conducted in the absence of any commercial or financial relationships that could be construed as a potential conflict of interest.
